# Prevalence and risk factors associated with HIV-1 infection among people who inject drugs in Dar es Salaam, Tanzania: a sign of successful intervention?

**DOI:** 10.1186/s12954-020-00364-5

**Published:** 2020-03-24

**Authors:** Samuel Lazarus Likindikoki, Elia John Mmbaga, Germana Henry Leyna, Kåre Moen, Neema Makyao, Mucho Mizinduko, Alex Ishungisa Mwijage, Diana Faini, Melkizedeck Thomas Leshabari, Dan Wolf Meyrowitsch

**Affiliations:** 1 Department of Epidemiology and Biostatistics, Muhimbili University of Health and Allied Sciences, 9 United Nations Road, P.O. Box 65001, Dar es Salaam, Tanzania; 2 Department of Psychiatry and Mental Health, Muhimbili University of Health and Allied Sciences, Dar es Salaam, Tanzania; 3 Department of Community Medicine and Global Health, Institute of Health and Society, University of Oslo, Oslo, Norway; 4 National AIDS Control Programme, Ministry of Health, Community Development, Gender, children and Elderly, Dodoma, Tanzania; 5 Department of Behavioral Sciences, Muhimbili University of Health and Allied Sciences, Dar es Salaam, Tanzania; 6 Department of Public Health, Global Health Section, University of Copenhagen, Copenhagen, Denmark

**Keywords:** Prevalence, HIV, Risk factors, Injection drug use, IBBS, Tanzania

## Abstract

**Background:**

Prevalence of HIV infection among people who inject drugs (PWID) has been reported to be higher than that of the general population. The present study aimed to estimate the prevalence of HIV infection and associated risk factors among PWID in Dar es Salaam, Tanzania, following the introduction of a comprehensive HIV intervention package (CHIP) for PWID in the country in 2014.

**Methods:**

We conducted an integrated bio-behavioral survey (IBBS) among PWID using respondent-driven sampling (RDS) in Dar es Salaam, Tanzania, between October and December 2017. Data on socio-demographic characteristics and risky behaviors were collected through face-to-face interviews. Blood samples were collected and tested for HIV infection. We accounted for weighting in the analyses, and logistic regression was performed to assess risk factors for HIV infection.

**Results:**

A total of 611 PWID (94.4% males and 5.6% females) with a median age of 34 years (IQR 29–38) were recruited. The overall prevalence of HIV infection was 8.7% (95% CI 6.5–10.9). The prevalence of HIV infections for males and females were 6.8% (95% CI 4.7–8.9%) and 41.2% (95% CI 23.7–58.6%) respectively. Adjusted weighted logistic regression analysis (WLRA) showed that being a female (aOR 19.1; 95% CI 5.9–61.8), injecting drugs for more than 10 years (aOR = 7.32; 95% CI 2.1–25.5) compared to 1 year or less and being 45 years or older (aOR = 34.22; 95% CI 2.4–489.5) compared to being 25 years or younger were associated with increased odds of HIV infection. Use of a sterile needle at last injection decreased odds of HIV infection (aOR = 0.3; 95% CI 0.1–0.8).

**Conclusions:**

The present study observed a decline in prevalence of HIV infections among PWID in Dar es Salaam (8.7%) compared to a previous estimate of 15.5% from an IBBS conducted in 2013. Despite the decrease, HIV prevalence remains high among PWID compared to the general population, and women are disproportionally affected. The decline may be possibly attributed to the on-going implementation of CHIP for PWID, highlighting the need for strengthening the existing harm reduction interventions by incorporating access to sterile needle/syringe and addressing the layered risks for women.

## 1. Background

Globally, it is estimated that there are 15.6 million people who inject drugs (PWID), and 30% of these are women [1]. In Tanzania, a national consensus size estimate from 2014 indicated the total number of people who inject drugs (PWID) to be 30,000 individuals [2]. Injection drug use poses health problems in both low-middle and high-income countries [3]. Among notable complications of injection drug use is the transmission of blood-borne infections, including HIV and viral hepatitis [4]. PWID are at risk of drug overdoses, including suicide related to drug use [5, 6]. In addition, they can be affected by other mental health problems [7]. Furthermore, injection drug use is also associated with stigma, rejection, and incarceration [8, 9]. PWID are disproportionally affected by HIV infection as compared to the general population. The global average prevalence of HIV infection among people who inject drugs is 17.8% [1]. Several studies conducted in Tanzania between 2010 and 2018 have reported that the prevalence of HIV infection among PWID ranges between 11.0 and 51.1% [2, 10–13]. These estimates are 3 to 11 times higher than the prevalence of HIV infection in the general population which is currently 4.5% [14]. In response to the high prevalence of HIV infection observed among PWID, the Government of Tanzania has rolled out a national comprehensive HIV intervention package (CHIP) for PWID in 2014. CHIP contains HIV intervention components such as biomedical interventions, social-behavioral interventions, HIV diagnosis, treatment and care, and management of co-infection and co-morbidities [15]. A total of six medication-assisted treatment (MAT) clinics have been established in mainland Tanzania along with several other harm reduction interventions including a pilot needle exchange program in one of the districts in Dar es Salaam. Since the rollout of the CHIP among key populations in Tanzania, no survey has been done to investigate the prevalence of HIV infection and the risk factors among PWID. The present study provides data on the prevalence of HIV infection and associated risk factors after the introduction of the CHIP in Tanzania.

## 2. Methods

### 2.1. Study design and setting

A cross-sectional survey was carried out between October and December 2017 in Dar es Salaam, the largest metropolitan city in Tanzania, with an estimated population of 6.7 million in 2020 [16]. This study was part of a broader integrated bio-behavioral survey that was conducted among key populations in Dar es Salaam. The city was chosen because of the high number of PWID [2] and a high prevalence of HIV infection [11, 12].

### 2.2. Sample size calculation

Sample size estimates were based on the measurement of precision of point estimates for the prevalence of HIV infection among PWID. The sample size and power calculation for this study was based on an estimated prevalence of 16% from the IBBS which was conducted in Dar es Salaam in 2014 [12]. The 95% confidence interval and a 5% standard error were used to calculate an appropriate sample size of 610 study participants.

### 2.3. Study population

The study participants were PWID aged 18 and above residing in Dar es Salaam. Residency was defined as having lived in the city for at least 12 months before the survey. PWID were included in the study if they had injected drugs in the past 3 months, presented with a valid study coupon, and consented to participate in the study.

### 2.4. Sampling procedures and recruitment

The study participants were recruited through respondent-driven sampling (RDS). RDS is a recruitment and sampling method that combines modified snowball sampling with a mathematical model that weights the sample to compensate for the fact that it was collected in a non-random way [17]. Research assistants in consultation with the study investigators purposefully selected and recruited five seeds (i.e., the initial survey participants). The selection was done so that the seeds came from multiple locations to improve access to potentially hidden networks of PWID. Subsequently, each seed person recruited a maximum of three other participants by giving them recruitment coupons, and this process continued so that the desired sample size and the statistical power were reached. Participants were screened for eligibility by a person who had injected drugs in the past and recovered after therapy. The screener used a standardized screening tool to ensure objectivity. Interviews were done at a private, safe location that was easily accessible to the participants. The screener verified eligibility for all study participants by looking for injection track marks and asking about knowledge of local terms, injection practices, and drug prices. The participants were only included in the study if they possessed a valid recruitment coupon. Each coupon was uniquely barcoded to link recruiters with recruitees, and the barcode was also used to connect participants with their consent forms, questionnaires as well as HIV test results. A total of 8000 Tanzanian shillings (3.50 USD) and 4000 Tanzanian shillings (1.75 USD) were paid to each participant for transport reimbursement and incentive for secondary recruitment, respectively. The amounts used for primary and secondary incentives were approved by the ethical review committee as the average travel costs to the study site.

### 2.5. Data collection training

A team of five research assistants with prior experience in research was recruited and provided with a two-day training on the study protocol, study instruments, research ethics, HIV testing, as well as the procedures for transportation and storage of biological samples. The team was oriented on the use of an open data kit (ODK) for data collection. ODK is a free and open-source data collection software that was customized for our research purpose. Additionally, the field team was trained on interviewing skills and sensitivity in working with key and vulnerable populations.

### 2.6. Data collection tool

A structured questionnaire was adapted from a previous IBBS which was done in 2013 among PWID in Dar es Salaam, Tanzania. The questionnaire was translated from the English to Kiswahili and then back translated to English to ensure face validity (a simple form of validity based on a subjective assessment). The version of the questionnaire used during the interviews was in Swahili, the language spoken by the majority of Tanzanians. The questionnaire was pre-tested and also evaluated with a group of PWID peer educators to ensure appropriate use of language and alignment with cultural and peer norms. Using handheld tablets, trained research assistants administered the questionnaire and collected data using an ODK, which were uploaded directly into the Muhimbili University of Health and Allied Sciences (MUHAS) server daily. The questionnaire gathered information on socio-demographic characteristics, injection practices, sexual risk behaviors, experience of violence, and alcohol use from the PWID participants. Also, Blood samples were also taken and tested for HIV infection.

## 3. Measures

### 3.1. Socio-demographic characteristics

Socio-demographic characteristics were gathered using a questionnaire to describe the sample population. It included age (continuous), gender (male or female), marital status (never married, married, separated/divorced/widowed), average level of income per month (continuous), employment status (none, self-employment, employed, informal employment), and levels of education (none, primary education, secondary education).

### 3.2. Injection practices and other risky behaviors for HIV infection

Injection practices and other HIV risk behaviors were measured as binary variables and included the following: frequency of alcohol consumption in the past one month, non-injection drug use other than alcohol in the last 3 months, ever shared needles, having shared needles in the past month, use of sterile needle at last injection, ever used male condoms, experience of physical violence (beaten) in the past 12 months, and forced to have sex in the past 12 months.

### 3.3. Biological data

Pre- and post-test counseling and HIV testing were performed by a nurse and a laboratory technician at the study site, and the national HIV testing algorithms were followed. All specimens were screened using SD Bioline HIV-1/2 3.0 test manufactured by Standard Diagnostics, Inc., Korea [18, 19], and positive samples were confirmed using Determine HIV1/2 test manufactured by Alere group of companies [20]. For discordant samples, the tiebreaker test was Unigold HIV test manufactured by Trinity Biotech, Ireland, and was used as a determining test [19].

### 3.4. Data management and statistical analysis

Data analysis was conducted using the RDS Analysis Tool (RDSAT) and STATA version 15.1. To be able to generate sample weight using RDSAT software package, participants were asked for the number of PWID they knew that they could recruit into the survey. They were also asked for the number of PWID they have seen during the last month, a number which was used in the analysis of data when weighing the participant’s network size. Per RDSAT data analysis procedures, data were weighted by calculating weight as an inverse of the participant’s network size and controlled for clustering by multiplying the weight by the sample size and then dividing by the sum of the weight. Categorical variables were summarized by calculating frequencies and percentages. The median and interquartile range were used to summarize the age variable. The chi-square test was used to compare differences in HIV infection by various categorical variables. Weighted logistic regression analysis was used to estimate bivariate of the associations between various risk factors and HIV infection. Only covariates with a *p* value threshold of < 0.25 in the bivariate analyses were included in the logistic regression model. Crude and adjusted odds ratios with 95% confidence intervals were reported. All the analyses were two-tailed, and a *p* value of less than 0.05 was considered statistically significant.

## 4. Results

### 4.1. Characteristic of study participants

The distribution of the socio-demographic characteristics of the study participants is shown in Table 1. A total of 611 PWID with a median age of 34 years (IQR 29–38) were recruited through eight recruitment waves. The majority of the study participants were males (94.4%). About half of those who took part were unmarried (52.9%), whereas 28.5% were divorced/separated or widowed. Most of the participants were within the age group 25–44 years (84.5%). About a third (30.4%) of the participants reported a monthly income above 200,000 Tanzanian shillings (about 86 USD), whereas 20.3% reported a monthly income of less than 50,000 shillings (less than 22 USD). The largest proportion of the study participants had completed primary school (67.6%). Almost three quarters (74.7%) of the study participants reported being self-employed.

**Table 1:** Descriptive characteristics of study participants (*N* = 611)

Characteristics	Number of participants (*n*)	Unweighted proportion (%)	Weighted proportion (%)
Sex			
Male	576	94.3	94.4
Female	35	5.7	5.6
Median age (IQR)	34 (29–38)		
Age (years)			
< 25	54	8.8	8.7
25–34	264	43.7	43.0
35–44	245	40.3	41.5
≥ 45	44	7.2	6.8
Completed educational level			
None	113	21.8	23.5
Primary school	418	68.4	67.6
Secondary school	60	9.8	8.9
Income per month (Tshs)			
< 50,000	116	19.0	20.3
50,000–120,000	145	23.7	25.7
120,000–200,000	95	15.6	17.6
> 200,000	255	41.7	30.4
Occupation			
None	24	3.9	3.6
Self-employment	450	73.6	74.7
Employed	100	16.4	16.3
Informal employment	37	6.1	5.4
Marital status			
Never married	323	52.9	52.7
Married	115	18.6	17.1
Separated/divorced/widowed	173	28.3	30.2

### 4.2. Risk factors for HIV infection

Table 2 shows the distribution of HIV risk factors. Almost one-third (29.0 %) of the study participants said they had used needles previously used by others in the past 1 month, while 41% reported ever have shared a needle with someone else. About one-third (35.0%) of the participants reported taking other non-injecting drugs other than alcohol. Just above half of the participants had injected drugs for 7 years or more, while 44% reported having injected drugs for more than 10 years. About one-fifth of the participants (18%) reported that they had not used a new sterile needle the last time they injected. A majority of the participants (69%) reported never having used alcohol. More than half of the participants (58%) had experienced both physical and sexual violence, while about 14% had experienced sexual violence in the past year. Three-quarters of the participants (75%) reported ever used a male condom.

**Table 2:** Weighted and unweighted proportion distribution of risk factors among PWID (*N* = 611)

Predictor	Number of participants (*n*)	Unweighted proportion (%)	Weighted proportion (%)
Used needle previous used by others in the past 1 month			
Yes	173	28.3	29.0
No	438	71.7	71.0
Ever shared a needle with someone else			
Yes	253	41.4	41.0
No	258	58.6	59.0
Taken any non-injected drugs other than alcohol in the last 3 months			
Yes	202	33.1	35.0
No	409	66.9	65.0
Duration of injection drugs			
< 1 year	47	7.7	9.0
1–3 years	108	17.7	17.0
4–6 years	117	19.1	29.0
7–9 years	76	12.4	12.0
10+ years	263	43.0	44.0
Used a new sterile needle last time injected			
Yes	503	82.3	82.0
No	108	17.7	18.0
Ever used a male condom			
Yes	455	74.5	75.0
No	156	25.5	25.0
Alcohol consumption			
Never	413	67.7	69.0
2–3 times a week	31	5.1	4.9
more times a week	39	6.4	5.1
2–4 times a month	127	20.8	20.0
Experience of both form of violence			
Yes	339	55.5	58
No	272	44.5	42
Experience of sexual violence			
Yes	91	14.9	14
No	520	85.1	86
Experience of physical violence			
Yes	226	37.0	35
No	385	63.0	65

### 4.3. Prevalence of HIV infection

The overall weighted prevalence of HIV infection was 8.7% (95% CI 6.4–11.8). Sex-disaggregated analysis indicated that among males and females, the prevalence of HIV infection was 6.8% (95% CI 4.7–8.9%) and 41.2% (95% CI 23.7–58.6%). Among participants, who were aged 45 and older, did not use a sterile needle at last injection, and had injected drug for 10 years or more, the prevalence of HIV was 22.4%, 16%, and 14.2%. Of all the three risk factors, people injected for 10 years or more have the least HIV prevalence.

### 4.4. Factors associated with HIV infection

Table 3 shows the results of bivariate and multivariate regression analyses. We found that female gender, older age, longer duration of injecting drug use, and using a sterile needle during the last injection were independently associated with HIV infection among PWID. Adjusted weighted analyses of risk factors for HIV infection indicated that being female (aOR = 19.1; 95% CI 5.9–61.8) was associated with increased odds of HIV infection compared to males. We also found that being aged 45 years or older (aOR = 34.2; 95% CI 2.4–489.5) was associated with an increased odds of HIV infection compared with those who were younger than 25 years. Persons who had injected drugs for more than 10 years had about seven times the odds of HIV infection as compared to persons who had injected drugs for less than a year (aOR = 7.3; 95% CI 2.1–25.5). However, using a sterile needle during the last injection was independently associated a 70% decreased odds of HIV infection (aOR = 0.3; 95% CI 0.1–0.8).

**Table 3:** Bivariate and multivariate analysis of predictors associated with HIV infection among PWID (*n* = 607)

Characteristics	Number of participants (weighted %)	Weighted % of HIV positive	Bivariate analysis		Multivariate analysis	
			cOR (95% CI)	*p* value	aOR (95% CI)	*p* value
Sex**						
Male	576 (94.4)	6.8	1		1	
Female	35 (5.6)	42.0	14.7 (4.9–43.1)	0.000*	19.1(5.9–61.8)	0.000*
Age group (years)						
< 25	54 (8.7)	2.9	1		1	
25–34	264 (43.0)	5.8	2.1 (0.3–16.8)	0.498	5.4(0.4–76.4)	0200
35–44	245 (41.5)	10.8	4.0 (0.5–30.9)	0.180	11.6(0.9–144.5)	0.057
**≥** 45	44 (6.8)	22.4	9.7 (1.1–86.9)	0.043*	34.2(2.4–489.5)	0.009*
Educational level						
None	113 (23.5)	10.8	1			
Primary education	418 (67.6)	7.4	0.7 (0.3–1.4)	0.273		
Secondary education	60 (8.9)	13.4	1.3 (0.4–3.6)	0.648		
Income per month (Tshs)						
< 50,000	116 (20.3)	10.0	1		1	
50,000–120,000	145 (25.7)	10.8	1.1 (0.4–2.8)	0.859	1.5 (0.5–4.5)	0.515
120,001–200,000	95 (17.6)	9.6	1.0 (0.4–2.6)	0.350	0.7 (0.2–2.3)	0.566
> 200,000	255 (30.4)	6.2	0.6 (0.3–1.4)	0.247	0.4 (0.2–1.9)	0.444
Occupation						
None	24 (3.6)	6.0	1			
Self employed	450 (74.7)	7.7	1.3 (0.2–11.0)	0.797		
Employed	100 (16.3)	6.5	1.1 (0.1–10.1)	0.946		
Informal employment	37 (5.4)	31.3	7.1 (0.8–63.1)	0.077		
Marital status						
Single	323 (52.7)	8.3	1			
Married partner	115 (17.1)	8.0	1.0 (0.4–2.4)	0.928		
Separated/Divorced	173 (30.2)	9.8	1.2 (0.56–2.6)	0.633		
Ever shared needle						
No	258 (41.0)	5.6	1		1	
Yes	253 (59.0)	13.4	2.6 (1.3–5.1)	0.005*	2.2(0.8–6.3)	0.148
Used needle previous used by others in the past 1 month						
No	438 (71.0)	6.8	1		1	
Yes	173 (29.0)	13.5	2.1 (1.08–4.24)	0.029*	0.7(0.2–2.0)	0.509
Taken any non-injected drugs other than alcohol in the last three months						
No	409 (65.0)	7.9	1			
Yes	202 (35.0)	10.4	1.4 (0.7–2.7)	0.382		
Duration injecting drugs (in years)						
< 1 year	47 (9.0)	2.9	1		1	
1–3 years	108 (17.0)	4.3	1.5 (0.2–8.8)	0.662	2.3 (0.5–10.9)	0.294
4–6 years	117 (29.0)	3.0	1.0 (0.1–7.4)	0.969	1.9 (0.3–10.0)	0.469
7–9 years	76 (12.0)	8.4	3.1 (0.5–17.3)	0.206	4.2 (0.9–19.0)	0.059
10+ years	263 (44.0)	14.2	5.5(1.2–25.8)	0.030*	7.3 (2.1–25.5)	0.002*
Used a new sterile needle last time injected						
No	108 (18)	16.4	1		1	
Yes	503 (82)	7.1	0.4 (0.2–0.8)	0.005*	0.3 (0.1–0.8)	0.020*
Ever used a male condom						
No	156 (25.0)	6.3	1		1	
Yes	455 (75.0)	9.5	1.6(0.7–3.4)	0.246	1.7 (0.6–4.7)	0.277
Alcohol consumption						
Never	413 (69.0)	8.0	1			
2–3 times a week	31 (4.9)	7.5	0.9 (0.3–3.4)	0.914		
4 or more times a week	39 (5.1)	10.9	1.4 (0.5–4.0)	0.515		
2–4 times a month	127 (20.0)	11.1	1.4 (0.6–3.4)	0.400		
Experience of both form of violence						
No	272 (42)	6.5	1			
Yes	339 (58)	11.8	1.9 (0.9–3.8)	0.064		
Experience of physical violence						
No	385 (65)	8.3	1			
Yes	226 (35)	9.7	1.2 (0.6–2.3)	0.616		
Experience of sexual violence						
No	520 (86)	6.9	1		1	
Yes	91 (14)	19.7	3.3 (1.6–6.7)	0.001*	1.2 (0.6–3.1)	0.718

Only covariates with a *p* value threshold of < 0.25 tin the bivariate analysis were included in the multivariate analysis

*AOR* adjusted odds ratio, *cOR* crude odds ratio, *CI* confidence interval

*A *p* value of 0.05 and below was considered statistically significant

**Unweighted Chi-square analysis was used to summarize of the percentage of HIV infection by sex

## 5. Discussion

With data from 611 PWID, we assessed the prevalence of HIV and associated risk factors among PWID in Dar es Salaam, Tanzania. We found that 8.7% of PWID residing in Dar es Salaam, Tanzania, were infected with HIV. This estimate is almost half of the prevalence reported by a similar IBBS conducted in 2013 in the same geographical area and by the same research team (15.5%). In 2011, the results of a survey among PWID attending outreach services in Dar e Salaam found HIV prevalence to be 51.1%, almost six times higher than the prevalence observed in the present study [11]. The decrease in HIV prevalence among PWID suggested by the findings of this study is in line with what has been observed in the general Tanzanian population over the past decade, during which the prevalence of HIV has decreased from 5.7% in 2008 to 4.7% in 2016 [14]. The underlying reasons for the observed decline could be multifactorial, e.g., mortality among HIV-infected PWID or practice of safer injecting and sexual practices among members of this population. It is also possible that the observed decline has occurred as a consequence of safer behaviors resulting from the rollout of the harm reduction services and comprehensive HIV interventions that were implemented in the country in 2012 and 2014 respectively. The latter included MAT services and the community-based and peer-led outreach services provided by several civil society organizations (CSOs) in the vicinity.

Globally, the trends of the HIV epidemic among PWID have not been well established. There are different reports indicating both an increase and a decrease in HIV infections in different parts of the world. Studies have shown a reduction in HIV infection among people who use drugs in some areas in the USA and Greece [21, 22], while other studies have shown increases, such as in Scotland [23]. Notable factors attributed to the observed reduction of HIV infection among PWID in Greece and Australia included the access to a combination of several prevention strategies such as medication-assisted treatment, antiretroviral treatment for HIV infection, and extensive needle/syringe exchange programs [22, 24].

We found that there is a gender disparity in HIV prevalence among PWID. Women had almost nineteen times higher the odds of HIV infection compared to men. As in the previous studies, we observed a higher prevalence of HIV infection among WWID than among their men counterparts [11, 12]. This gender disparity in HIV infection may exist because women who inject drugs have multiple layers of HIV infection risk. Women who inject drugs may have additional risks related to unsafe sexual practices, such as multiple concurrent sexual partnerships, having an injection partner who is also a sexual partner, and engaging in commercial sex to be able to purchase drugs [25].

The duration of injecting drug use and the age of PWID were found to be associated with HIV infection in the present study. However, there is a potential collinearity between age of the PWID and the year spent injecting drugs. This may mean that older PWID are more likely to have spent many years injecting drugs and make it possible that both variables measure the same construct (i.e., duration of exposure to drug injection). Having injected drugs for more than 10 years and being aged 45 years or older significantly increased the odds of HIV infection. This finding is similar to other studies among PWID [26]. The cumulative risk resulting from older age and a long duration of exposure to HIV risk behaviors may explain this association. Additionally, the availability of HIV prevention and treatment services for PWID which were established less than 10 years ago could also partly explain these findings. This finding may mean that before the rollout of harm reduction services, PWID were more likely to engage in unsafe injection practices and also other behaviors associated with HIV risk that could explain the increased odds of HIV infection among the older PWID and those who used drugs for longer duration. Studies in Sub Saharan Africa including from Tanzania have shown that the coverage of harm reduction intervention is very low, ranging from 20 to 40% [2]. By the end of 2015, only 2500 out of nearly 30,000 PWID were on MAT in Tanzania [2]. It is imperative that the efforts to scale up harm reduction interventions are increased to prevent continued HIV risk exposure among PWID.

This study found that the use of clean syringes and needles was independently associated with decreased odds of HIV infection among PWID. These findings concur with other studies that have found needled and syringe programs (NSPs) to be effective in reducing HIV infection [24, 27]. It is important to carefully evaluate the current National Guideline for a Comprehensive HIV Package for Key Population and strengthen the NSPs as part of harm reduction for PWID in Tanzania.

## 6. Limitations

There are some limitations to this study. First, this is a cross-sectional study which provides a snapshot of the magnitude (prevalence) of HIV infection among PWID. However, it does not allow for evaluation of the temporal relationships between HIV infection and the predictors of interest. Secondly, there is a possibility of desirability bias due to the collection of sensitive information related to injecting and sexual behaviors. Thirdly, there is a potential for selection bias inherent in the respondent-driven sampling which is a non-probabilistic sampling approach. To mitigate this, seeds were selected in such a way that they could represent the diversity of PWID, including gender (male and female), various age groups, and analysis, and took into account network size and used special software (RDSAT) designed for this kind of recruitment. Lastly, out of 611 participants, 35 were WWID constituting only 5.6% of the study sample. Despite the fact that previous studies in the same city have also reported fewer women who inject drugs and higher HIV infection rates, the lower proportion of women in this study may have biased gender comparison [12]. Difficulties in recruiting WWID in Tanzania context can be explained by the idea that, WWID are subjected to increased risk of physical violence (such as assaults and robbery) as well as stigma and discrimination which in turn generate mistrust against health-related activities and eventually push them in hiding and being less accessible through the study’s RDS [28]. In addition, some WWID are engaging in commercial sex work which happens mostly during the night time and hence make it difficult to be accessed during the day time when RDS was taking place.

## 7. Conclusion

The results from this study suggest that the prevalence of HIV among PWID in Dar es Salaam has decreased considerably in the past 4 years. Despite the declining trends of HIV infection for both the general population and among PWID, the risk of being HIV positive for PWID is almost twice that of people in the general population. The decline in the prevalence of HIV could be attributed to increased access to comprehensive HIV interventions for key populations in Dar es Salaam, which includes harm reduction intervention and access to HIV testing, care, and treatment. The results may be a sign of successful interventions that are helping to realize the global targets of 90-90-90 and three zeros [29]. Comprehensive HIV interventions should be rolled out across the country, especially in the areas where there is a substantial number of PWID.

## Data Availability

Data from this study can be accessed from the corresponding author upon reasonable request.

## Abbreviations

AIDS: Acquired immune deficiency syndrome
AOR: Adjusted odds ratio
COR: Crude odds ratio
IBBS: Integrated bio-behavioral survey
IQR: Interquartile range
MAT: Medication assisted treatment
NSPs: Needled and syringe programs
ODK: Open data kit
PWID: People who inject drugs
RDS: Respondent-driven sampling

## References

[1] Mathers BM, Degenhardt L, Phillips B, Wiessing L, Hickman M, Strathdee SA, Wodak A, Panda S, Tyndall M, Toufik A, Mattick RP (2008). 2007 Reference Group to the UNon HIV and Injecting Drug Use. Global epidemiology of injecting drug use and HIV among people who inject drugs: a systematic review. Lancet..

[2] Dutta, Barker C, Makyao N A. Consensus estimates on key population size and HIV prevalence in Tanzania. National AIDS Control Programme. 2014. 1–34 p. Available from:https://www.healthpolicyproject.com/pubs/391_FORMATTEDTanzaniaKPconsensusmtgreport.pdf.

[3] Csete J, Kamarulzaman A, Kazatchkine M, Altice F, Balicki M, Buxton J, Cepeda J, Comfort M, Goosby E, Goulão J, Hart C, Kerr T, Lajous AM, Lewis S, Martin N, Mejía D, Camacho A, Mathieson D, Obot I, Ogunrombi A, Sherman S, Stone J, Vallath N, Vickerman P, Zábranský T, Beyrer C (2016). Public health and international drug policy. Lancet..

[4] Gordon RJ, Lowy FD (2005). Bacterial infections in drug users. N Engl J Med..

[5] Dragisic T, Dickov A, Dickov V, Mijatovic V (2015). Drug addiction as risk for suicide attempts. Mater Sociomed..

[6] Mathers BM, Degenhardt L, Bucello C, Lemon J, Wiessing L, Hickman M (2013). Mortality among people who inject drugs: a systematic review and meta-analysis. Bull World Health Organ..

[7] Wu LT, Blazer DG (2014). Substance use disorders and psychiatric comorbidity in mid and later life: a review. Int J Epidemiol..

[8] DeBeck K, Cheng T, Montaner JS, Beyrer C, Elliott R, Sherman S, Wood E, Baral S (2017). HIV and the criminalisation of drug use among people who inject drugs: a systematic review. Lancet HIV.

[9] Topp L, Iversen J, Conroy A, Salmon AM, Maher L (2008). Collaboration of Australian NSPs. Prevalence and predictors of injecting-related injury and disease among clients of Australia’s needle and syringe programs. Aust N Z J Public Health..

[10] Williams ML, McCurdy SA, Bowen AM, Kilonzo GP, Atkinson JS, Ross MW, Leshabari MT (2009). HIV seroprevalence in a sample of Tanzanian intravenous drug users. AIDS Educ Prev.

[11] Cassian Nyandindi, Jessie Mbwambo, Sheryl McCurdy (2011). HIV serostatus, Hepatitis C and depression among injecting drug users in Kinondoni municipality Dar Es Salaam, Tanzania. Mmed Psychiatry dissertation https://ir.muhas.ac.tz:8080/jspui/handle/123456789/20.

[12] Mmbaga EJ, Moen K, Makyao N, Leshabari M (2017). Prevalence and predictors of human immunodeficiency virus and selected sexually transmitted infections among people who inject drugs in Dar es Salaam, Tanzania: A New Focus to Get to Zero. Sex Transm Dis..

[13] Khatib A, Matiko E, Khalid F, Welty S, Ali A, Othman A, Haji S, Dahoma M, Rutherford G (2017). HIV and hepatitis B and C co-infection among people who inject drugs in Zanzibar. BMC Public Health..

[14] WHO. United Republic of Tanzania: HIV Country Profile: 2016. Who/Hiv/201759 [Internet]. 2017;2016–7. Available from:https://www.who.int/hiv/data/Country_profile_United_Rep_of_Tanzania.pdf?ua=1www.who.int/hiv/data/Country_profile_United_Rep_of_Tanzania.pdf?ua=1.

[15] National Guideline For Comprehensive Package Of HIV Interventions For Key Populations. Available from: https://www.hivsharespace.net/sites/default/files/resources/7.%202014%20Tanzania_KP_Comprehencive_Guideline_sept_29th_2014%20%281%29.pdf.

[16] Nations U. World Urbanization Prospects The 2018 Revision. 2018; Available from: https://population.un.org/wup/Country-Profiles/.

[17] RDS reference.pdf. Available from: http://www.respondentdrivensampling.org.

[18] Shimelis T, Tadesse E (2015). The diagnostic performance evaluation of the SD BIOLINE HIV/syphilis Duo rapid test in southern Ethiopia: a cross-sectional study. BMJ Open.

[19] Crucitti T, Taylor D, Beelaert G, Fransen K, Van Damme L (2011). Performance of a rapid and simple HIV testing algorithm in a multicenter phase III microbicide clinical trial. Clin Vaccine Immunol.

[20] van den Berk GE, Frissen PH, Regez RM, Rietra PJ (2003). Evaluation of the rapid immunoassay determine HIV 1/2 for detection of antibodies to human immunodeficiency virus types 1 and 2. J Clin Microbiol.

[21] Mitsch AJ, Hall HI, Babu AS (2016). Trends in HIV infection among persons who inject drugs: United States and Puerto Rico, 2008-2013. Am J Public Health..

[22] Sypsa V, Psichogiou M, Paraskevis D, Nikolopoulos G, Tsiara C, Paraskeva D, Micha K, Malliori M, Pharris A, Wiessing L, Donoghoe M, Friedman S, Jarlais DD, Daikos G, Hatzakis A (2017). Rapid decline in HIV incidence among persons who inject drugs during a fast-track combination prevention program after an HIV outbreak in Athens. J Infect Dis..

[23] Ragonnet-Cronin M, Jackson C, Bradley-Stewart A, Aitken C, McAuley A, Palmateer N, Gunson R, Goldberg D, Milosevic C, Leigh Brown AJ (2018). Recent and rapid transmission of HIV among people who inject drugs in Scotland revealed through phylogenetic analysis. J Infect Dis..

[24] Wodak A, Maher L (2010). The effectiveness of harm reduction in preventing HIV among injecting drug users. N S W Public Health Bull..

[25] Azim T, Bontell I, Strathdee SA (2015). Women, drugs and HIV. Int J Drug Policy..

[26] Montain J, Ti L, Hayashi K, Nguyen P, Wood E, Kerr T (2016). Impact of length of injecting career on HIV incidence among people who inject drugs. Addict Behav.

[27] Kerr T, Small W, Buchner C, Zhang R, Li K, Montaner J, Wood E (2010). Syringe sharing and HIV incidence among injection drug users and increased access to sterile syringes. Am J Public Health..

[28] Zamudio-Haas S, Mahenge B, Saleem H, Mbwambo J, Lambdin BH (2016). Generating trust: Programmatic strategies to reach women who inject drugs with harm reduction services in Dar es Salaam. Tanzania. Int J Drug Policy..

[29] UNAIDS. 90-90-90 An ambitious treatment target to help end the AIDS epidemic. United Nations. 2014; Available from: https://www.unaids.org/sites/default/files/media_asset/90-90-90_en_0.pdf.

